# Polycythemia vera treatment algorithm 2018

**DOI:** 10.1038/s41408-017-0042-7

**Published:** 2018-01-10

**Authors:** Ayalew Tefferi, Alessandro M. Vannucchi, Tiziano Barbui

**Affiliations:** 1 Division of Hematology, Department of Medicine, Mayo Clinic, Rochester, MN USA; 2 Department of Experimental and Clinical Medicine, CRIMM, Center Research and Innovation of Myeloproliferative Neoplasms, Azienda Ospedaliera Universitaria Careggi, University of Florence, Florence, Italy; 3 Research Foundation, Papa Giovanni XXIII Hospital, Bergamo, Italy

## Abstract

Recently reported mature survival data have confirmed the favorable prognosis in polycythemia vera (PV), with an estimated median survival of 24 years, in patients younger than age 60 years old. Currently available drugs for PV have not been shown to prolong survival or alter the natural history of the disease and are instead indicated primarily for prevention of thrombosis. Unfortunately, study endpoints that are being utilized in currently ongoing clinical trials in PV do not necessarily target clinically or biologically relevant outcomes, such as thrombosis, survival, or morphologic remission, and are instead focused on components of disease palliation. Even more discouraging has been the lack of critical appraisal from “opinion leaders”, on the added value of newly approved drugs. Keeping these issues in mind, at present, we continue to advocate conservative management in low-risk PV (phlebotomy combined with once- or twice-daily aspirin therapy) and include cytoreductive therapy in “high-risk” patients; in the latter regard, our first, second, and third line drugs of choice are hydroxyurea, pegylated interferon-α and busulfan, respectively. In addition, it is reasonable to consider *JAK2* inhibitor therapy, in the presence of protracted pruritus or markedly enlarged splenomegaly shown to be refractory to the aforementioned drugs.

## Introduction

Polycythemia vera (PV) is currently classified by the World Health Organization (WHO) classification system under the major category of myeloproliferative neoplasms (MPN)^1^. Although the WHO MPN category includes seven subcategories, the term “MPN” usually refers to the three *JAK2* mutation-enriched clinicopathologic entities: PV, essential thrombocythemia (ET) and primary myelofibrosis (PMF)^1^. PV and its sister diseases constitute stem cell-derived clonal myeloproliferation that is characterized by three mutually-exclusive “driver” mutations: *JAK2*, *CALR*, and *MPL*, with respective distribution frequency of ~99, 0, and 0% for PV, 55, 22, and 3% for ET and 65, 20 and 7% for PMF^2^. The most frequent MPN-associated *JAK2* mutation is the exon 14 *JAK2*V617F, which is responsible for almost all the *JAK2* mutations in ET and PMF, and 97% of those seen in PV; the remainder 3% of *JAK2* mutations in PV are spread across exons 12, 13, and 14^3,4^.

Diagnosis of PV often requires the presence of a *JAK2* mutation, in addition to documentation of increased hemoglobin/hematocrit, to a threshold level established by the 2016 World Health Organization (WHO) revised criteria (>16.5 g/dL/49% for males and >16 g/dL/48% for females)^1^. In addition, bone marrow morphologic assessment is encouraged, in order to distinguish PV from *JAK2*-mutated ET^5-7^ and obtain cytogenetic information, which has recently been shown to be prognostically relevant^8–10^. Clinical features in PV include mild-to-moderate degree of splenomegaly, mild-to-moderate degree of constitutional symptoms, including fatigue and pruritus, symptoms of hyperviscosity, leukocytosis, thrombocytosis, microvascular symptoms (e.g., headaches, lightheadedness, visual disturbances, atypical chest pain, erythromelalgia, paresthesia), thrombotic and bleeding complications, and risk of leukemic transformation or fibrotic progression^11^.

Current treatment in PV has not affected the natural history of the disease in regards to overall, leukemia-free or myelofibrosis-free survival, but thrombosis-free survival has been positively affected by treatment with phlebotomy^12^, aspirin^13^ and cytoreductive drugs^11^. In the latter regard, the most popular and evidence-supported cytoreductive agent is hydroxyurea, while busulfan has been effectively and safely utilized for an even longer period^11,14^. More recently, interferon (IFN)-α and ruxolutinib (a JAK1 and JAK2 inhibitor) have been introduced to the therapeutic armamentarium, without controlled evidence of superiority over the older drugs and documentation of safety during long-term use. In the current review, we provide a risk-adapted treatment algorithm in PV, including critical assessment of the currently available cytoreductive agents.

## Risk-adapted treatment algorithm in polycythemia vera

### Survival and complications rates

Survival in PV is inferior to that of ET but superior to that of PMF, with estimated medians of 14, 20, and 6 years, respectively;^15^ the corresponding figures for patients younger than age 60 years are 24, 33, and 15 years^15^. Life-expectancy in all three MPN is significantly worse than that of the age- and sex-matched general population^15^. These observations are similar to those from a large population-based study of 9,384 patients^16^. The major life-threatening complications in PV are leukemic transformation, fibrotic progression and thrombosis, with incidence ranges of 5.5–18.7, 6–14, and 6–17%, respectively, over the course of 15, 15, and 3 years^17,18^.

### Risk factors for survival and predictors of leukemic or fibrotic progression

In the largest international study of 1545 patients with PV, independent risk factors for overall survival included age > 61 years, leukocyte count > 10.5 × 10(9)/L, venous thrombosis and abnormal karyotype and for leukemia-free survival age >61 years, leukocyte count >15 × 10^9^/L and abnormal karyotype;^10^ median survivals were 23 and 9 years, in the absence or presence of the first two risk factors; these observations were validated by another population-based study of 327 patients^19^.

The prognostic relevance of karyotype in PV was recently confirmed by subsequent Mayo Clinic and MD Anderson Cancer Center (MDACC) reports;^8,9^ in both studies, abnormal karyotype was reported in approximately 20% of patients (+9, +8, and 20q− being the most frequent) and adversely affected overall and transformation-free survival. Other genetic alterations, revealed by next generation sequencing, occur in the majority of patients with PV, and include *TET2* (22% frequency), *ASXL1* (12%), and *SH2B3* (9%) mutations^20^. Some of these mutations, in particular *ASXL1, SRSF2*, or *IDH2*, have been shown to adversely impact overall and transformation-free survival; median survival of patients with and without adverse mutations was 7.7 vs. 16.9 years, respectively^20^.

### Incidence of thrombosis and bleeding

The European Collaboration on Low-dose Aspirin in PV (ECLAP) recruited 1638 patients with Polycythemia Vera Study Group (PVSG)-defined PV, at variable times from initial diagnosis, and reported thrombosis history at time of recruitment in 39% (29% arterial and 14% venous) of the patients^21,22^. After a median follow-up of 2.8 years, 14% of the patients experienced cardiovascular events (incidence of 5.5 events/100 persons/year; 6.95 and 2.52 in high and low-risk patients, respectively) and thrombosis accounted for the main cause of death (41%). Bleeding history in the ECLAP study was 8.1% at time of study entry and 2.9% during follow-up.

In a more recent retrospective study of 1545 patients with WHO-defined PV, conducted by the International Working Group for MPN Research and Treatment (IWG-MRT), thrombosis history at diagnosis was documented in 23% of the patients and included 16% arterial and 7.4% venous events^23^. These figures were lower than those described above for the ECLAP study but similar to those reported by the Cytoreductive Therapy in PV (CYTO-PV) study (17% arterial and 12% venous), which included patients with WHO-defined PV^12^. The rate of post-diagnosis vascular events in the IWG-MRT study, after a median follow-up of 6.9 years, was 21% (2.62% patients/year), including 12% arterial and 9% venous events; additional analysis revealed 21% and 23% incidence of thrombosis history at diagnosis, in patients diagnosed before or after 2005, respectively;^18^ the corresponding thrombosis rates after median follow-up of 2.5–3.5 years from diagnosis were 10%/17% in low/high-risk patients diagnosed before 2005 and 6%/7% for those diagnosed after 2005^18^.

### Risk factors for thrombosis and current risk stratification

Risk factors for thrombosis, in the aforementioned ECLAP study, were age >65 years, thrombosis history, hypertension, tobacco use and congestive heart failure;^21,22^ subsequent observations from the same study also identified leukocyte count >15 × 10^9^/L compared to <10 × 10^9^/L, but not hematocrit level or platelet count^24^, as a risk factor for thrombosis, especially myocardial infarction^25^. The potential contribution of increased leukocyte count to thrombosis in PV was also highlighted in the context of the CYTO-PV study^26^ and recurrent thrombosis, especially in patients younger than age 60 years^27,28^. In the IWG-MRT study, arterial and venous thromboses were the main risk factors for recurrent arterial or venous vascular events, respectively^23^. In addition, history of hypertension predicted arterial thrombosis and advanced age (≥65 years) venous thrombosis. A more recent study has suggested that arterial hypertension might be a significant risk factor for thrombosis, even in low-risk patients^29^. Another study suggested that PV patients with bone marrow fibrosis might be at a lower risk for thrombosis^30^.

**Fig. 1:**
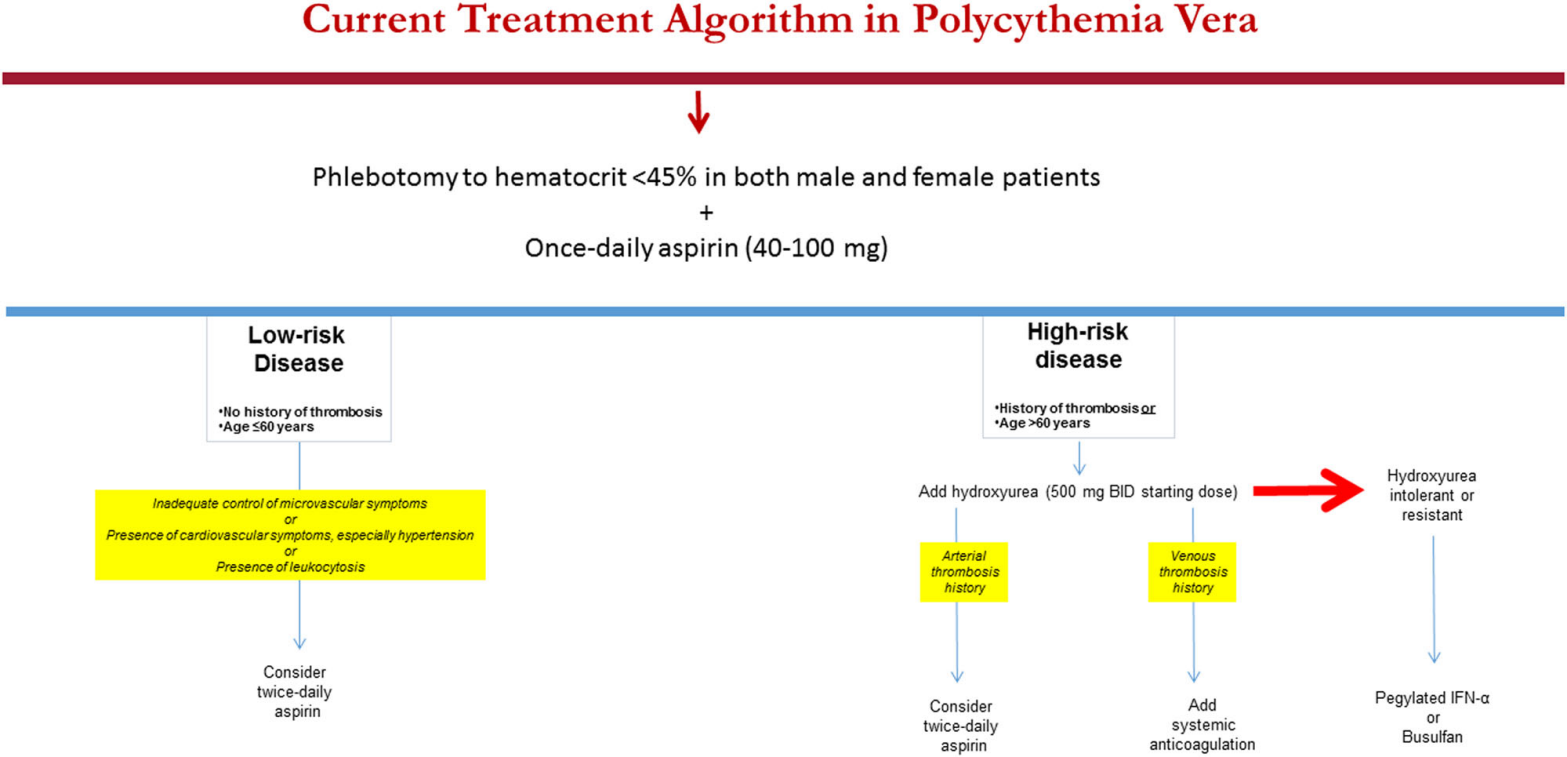
Current treatment algorithm in Polycythemia vera

On the basis of the above, we consider PV patients with thrombosis history to be at a significantly higher risk for recurrent thrombosis; in this regard, it is therapeutically relevant to distinguish patients with arterial vs venous thrombosis history (Fig. 1). The above-described studies also identify advanced age as an independent risk factor for thrombosis in PV and, accordingly, patients with either thrombosis history or advanced age are currently classified as having “high-risk” disease, while the absence of both risk factors is required for “low-risk” disease classification (Fig. 1). In addition, although not included in our current risk stratification scheme, we take the presence of hypertension and leukocytosis into consideration, when deciding treatment in certain circumstances (Fig. 1).

### Risk-adapted therapy: low-risk disease

Prior to the introduction of phlebotomy as a treatment modality in PV, reported median survivals for untreated PB were less than 2 years and attributed to excess death from thrombotic complications^31^. The anti-thrombotic value of aggressive phlebotomy in PV was reinforced by a randomized study that showed the benefit of keeping the hematocrit below 45%^12^. Controlled studies have also confirmed the additional anti-thrombotic value of low-dose aspirin in PV, among all risk categories^13^. Accordingly, we recommend aspirin therapy (81–100 mg once-daily) + phlebotomy with a target hematocrit of 45%, in all male and female patients with PV, regardless of risk status (Fig. 1). In addition, the emerging data from laboratory studies and clinical observations suggest that the increased platelet turnover in MPN results in suboptimal 24-hour suppression of thromboxane-A2 synthesis by once-daily dosing;^32,33^ therefore, we consider twice-daily dosing in low-risk patients whose microvascular symptoms are not adequately controlled with once-daily dosing or who are at high-risk for arterial thrombosis, including those with cardiovascular risk factors (especially hypertension) and leukocytosis (Fig. 1).

Most recently, two studies have re-visited the issue of the frequency of phlebotomy and thrombosis risk in PV, based on older polycythemia vera study group data that suggested increased risk of thrombosis in the first 3 years of treatment, in patients treated with phlebotomy alone^34,35^. In the first observational study^35^, the authors showed an association between requiring 3 or more phlebotomies per year and increased risk of thrombosis, despite concomitant treatment with hydroxyurea. This observation was not confirmed by the more robust second study that utilized data from a controlled study and implicated uncontrolled hematocrit level instead of frequency of phlebotomy as the culprit;^34^ furthermore, there is additional evidence that suggests the contribution of leukocytosis to the increased risk of thrombosis in patients with inadequate control of hematocrit^26^.

### Risk-adapted therapy: high-risk disease

Our current recommendations on the management of high-risk PV are based on both controlled and large retrospective and single arm prospective studies (Fig. 1). The PVSG conducted the first controlled study in PV (1967–1974) that compared treatment with phlebotomy alone or in combination with either oral chlorambucil or intravenous radioactive phosphorus (P32). The results revealed accelerated leukemic transformation and shortening of survival in patients receiving chlorambucil of P32^36,37^. During the same time period (1967–1978), the European Organization for Research on Treatment of Cancer (EORTC) conducted another randomized study in PV comparing oral busulfan with P32 and showed a survival advantage for busulfan^38^.

Other randomized studies in PV compared hydroxyurea with pipobroman (shorter survival and an increased risk of leukemic transformation with pipobroman therapy)^39,40^, P32 alone or with hydroxyurea (no difference in survival but the combination treatment was associated with increased leukemic transformation)^41^, and P32 + phlebotomy vs phlebotomy + high-dose aspirin (900 mg/day) in combination with dipyridamole (225 mg per day) (the addition of anti-platelet agents increased the risk of gastrointestinal bleeding)^42^. However, in a subsequent study by ECLAP, a lower dose of aspirin (100 mg daily), compared to placebo, was not associated with excessive bleeding and was shown to reduce thrombosis risk^13^.

The above-outlined observations on hydroxyurea treatment for PV were further supported by several other uncontrolled studies, including a PVSG study where the drug was associated with lower incidences of thrombosis, compared to a historical cohort treated with phlebotomy alone (6.6% vs 14% at 2 years), and leukemic transformation, compared to a historical control treated with either chlorambucil or P32 (5.9 vs. 10.6 vs. 8.3%, respectively, in the first 11 years of treatment)^43^. Several other uncontrolled studies have since confirmed the lack of association between hydroxyurea treatment and leukemic transformation with reported incidence range of 1–5.6%^44–46^. Pegylated IFN-α, as initial therapy for PV, has also been shown to be safe and effective, with reported complete hematologic and molecular response rates of 76–95 and 18%, respectively;^47,48^ overall and complete hematologic remission rates from an ongoing phase-3 study were 81% and 19%^49^.

On the bais of the above observations, our first-line cytoreductive drug of choice in PV is hydroxyurea (Fig. 1). This, however, does not undermine the possible value of IFN-α as first-line therapy, as an alternative to hydroxyurea, but follow-up time and number of patients treated so far with IFN-α are not adequate enough to justify such a measure at the present time. Our reservation, in this regard, is consistent with the results of interim analysis from an ongoing phase-3 study in PV and ET that suggested similar efficacy but higher toxicity events with pegylated IFN-α, compared to hydroxyurea^49^. Similarly, the relevance to disease outcome of molecular remissions seen in some PV patients treated with either IFN-α^47,48,50^ or busulfan^51^ is currently unclear.

Finally, there is evidence from observational studies that the use of oral anticoagulants, as well as that of aspirin therapy, prevents recurrent venous thrombosis in PV^28,52^. In one of the few studies that addressed the issue of recurrent thrombosis in PV, the authors were able to demonstrate the anti-thrombotic value of cytoreductive therapy in combination with either anti-platelet agents or systemic anticoagulation^28^. Furthermore, the study identified specific value for systemic anticoagulation, aspirin therapy and cytoreduction, in the prevention of venous thrombosis, cerebral and coronary events, respectively^28^. Interestingly, aspirin therapy was also associated with lower risk of venous thrombosis^28^. These observations were taken into consideration in formulating our treatment recommendations for high-risk disease (Fig. 1).

### Treatment options for hydroxyurea intolerant or refractory disease

We currently consider three drugs, as second-line therapy for PV: pegylated IFN- α, busulfan and ruxolutinib. Among the three, long-term safety data are available and considered acceptable for pegylated IFN-α and busulfan (as discussed above). Also, these two drugs, compared to ruxolutinib, display broader activity against clonal myeloproliferation and display better quality of response, including the ability to induce molecular remission^47,48,50,51^, although the clinical relevance of the latter is debatable. Furthermore, there is already extensive experience in the use of both drugs as initial therapy for PV, with results that appear to be comparable to that of hydroxyurea, as discussed above in the previous section.

In addition to the experience from the aforementioned randomized studies (see above), favorable outcome has also been reported in single arm studies using busulfan as initial therapy;^53,54^ in 65 busulfan-treated patients with PV followed between 1962 and 1983, median survival was 19 years in patients whose disease was diagnosed before age 60 years and only 2 patients (3.5%) developed acute leukemia^53^. Busulfan has also been shown to induce durable hematologic response in the majority and molecular response in a minority of patients who are intolerant or resistant to hydroxyurea therapy^55–57^. The concern regarding leukemogenicity of busulfan is unfounded and not supported by controlled evidence; in a large international study of over 1500 patients with PV, neither busulfan nor IFN- α were implicated as being leukemogenic^10^. Incidentally, busulfan expresses less DNA/RNA binding, compared to other alkylating agents, no inter- or intra-strand DNA binding and no immunosuppression^14^.

In a randomized study, ruxolitinib was compared to best available therapy, in hydroxyurea-resistant or intolerant PV with splenomegaly;^58^ treatment with ruxolutinib produced higher rates of hematocrit control (60 vs 20%), ≥ 35% reduction in spleen volume (38 vs 1%) and symptom control (49 vs 5%). However, ruxolutinib therapy was associated with higher rate of herpes zoster infection (6 vs 0%) and showed limited evidence of disease-modifying activity, with complete hematologic remission rate of only 24% and complete molecular remission rate of <2%^59^. Furthermore, the study was not designed to address clinically relevant endpoints in PV such as thrombosis-free, leukemia-free or myelofibrosis-free survival or bone marrow morphologic remission. Also, resistance to hydroxyurea treatment in PV might reflect more aggressive disease biology that warrants disease-modifying rather than palliative treatment strategy^60^. Finally, because of their relatively long survival, ruxolutinib-treated patients with PV are vulnerable to drug-induced immunosuppression and opportunistic infections, including reactivation of viral diseases^61^.

### Management of pruritus

Pruritus, sometimes aquagenic, is one of the most annoying, but not necessarily life-threatening complications of PV. In fact, in a recent large international study of 1545 patients with PV, presence of pruritus was independently associated with longer survival^10^. Among 441 German patients with PV, 301 (68%) reported pruritus (severe and unbearable in 15%), occurring in the majority prior to the diagnosis of PV^62^. In our own experience involving 418 patients seen at the Mayo Clinic, pruritus at diagnosis was documented in 31% and was associated with a lower rate of arterial thrombosis and higher *JAK2*V617F allele burden^63^. The latter observation was confirmed by others as well. Treatment options for PV-associated pruritus include antihistamines^64^, selective serotonin reuptake inhibitors (SSRIs)^65^, danazol^66^, IFN-α^67^, narrow-band ultraviolet B phototherapy^68^, photochemotherapy with psoralen and ultraviolet A light (PUVA)^69^, and JAK2^70^ or mTOR inhibitors^71^. Amongst these, we recommend initial therapy with antihistamines and SSRIs, followed by IFN-α therapy for more severe and refractory cases, and reserve treatment with JAK2 inhibitors for high-risk patients with IFN-α-resistant pruritus.

### Management during pregnancy

There are few published data on the occurrence and outcome of pregnancy in PV patients^72–74^. Therefore, our recommendations in this regard are mostly extrapolated from the experience in ET, which is consistent with the management strategy outlined in the limited series of anecdotal reports on PV. As is the case with ET, there appears to be increased miscarriage rates but otherwise mostly uneventful course and successful outcome^72–74^. Accordingly, we do not consider pregnancy to be contraindicated in women with PV and advise conservative management with once-daily aspirin therapy and phlebotomy to be adequate in “low risk” women, while we recommend the addition of pegylated IFN- α for high-risk disease^75^. In addition, patients should be closely monitored for pregnancy-induced hypertension.

## Conclusions

Patients with PV should look forward to long and productive life and avoid exposure to new drugs whose long-term consequences are not known and might include acceleration of clonal degeneration into acute myeloid leukemia or myelofibrosis. This is not only of theoretical concern and has happened before to PV patients treated with chlorambucil, radiophosphorus or pipobroman^37,39^ and to ET patients treated with anagrelide^76^. Decades of experience with hydroxyurea and busulfan, for the treatment of PV or ET, has not produced controlled evidence that implicates either one of these two drugs as being leukemogenic or immunosuppressive. Our concern lies with the newer drugs, including IFN-α^77^ and ruxolutinib^78,79^, neither of which has been shown to be superior or safer than conventional therapy, in a controlled study with clinically relevant endpoints (e.g., survival, thrombosis or bone marrow morphologic remission). Furthermore, neither IFN-α nor ruxolutinib induces morphologic or cytogenetic remission in PV or has been shown to alter the natural history of the disease; of note, the clinical relevance of IFN-α-induced suppression of *JAK2*V617F allele burden, which is seen in a small minority of patients^47^, and also documented with busulfan therapy^51^, is unclear. Therefore, it is incumbent upon the MPN community of physicians and scientists to dig deeper into more precise pathogenetic mechanisms and identify targetable disease-specific pathways. In other words, our patients need drugs with anti-tumor activity and do not have to settle for symptomatic relief.
